# Preparation, Evaluation, and Bioinformatics Study of Hyaluronic Acid-Modified Ginsenoside Rb1 Self-Assembled Nanoparticles for Treating Cardiovascular Diseases

**DOI:** 10.3390/molecules29184425

**Published:** 2024-09-18

**Authors:** Lixin Du, Yifei Xiao, Qidong Wei, Zhihua Guo, Ya Li

**Affiliations:** 1 School of Pharmacy, Hunan University of Chinese Medicine, Changsha 410208, China; 20223723@stu.hnucm.edu.cn (L.D.); 20213659@stu.hnucm.edu.cn (Y.X.); 20233763@stu.hnucm.edu.cn (Q.W.); 2 School of Chinese Medicine, Hunan University of Chinese Medicine, Changsha 410208, China; 004294@hnucm.edu.cn

**Keywords:** ginsenoside Rb1, self-assembled nanoparticles, inflammation, oxidative stress, cardiovascular diseases, bioinformatics

## Abstract

(1) Objective: To optimize the preparation process of hyaluronic acid-modified ginsenoside Rb1 self-assembled nanoparticles (HA@GRb1@CS NPs), characterize and evaluate them in vitro, and investigate the mechanism of action of HA@GRb1@CS NPs in treating cardiovascular diseases (CVDs) associated with inflammation and oxidative stress. (2) Methods: The optimal preparation process was screened through Plackett–Burman and Box–Behnken designs. Physical characterization of HA@GRb1@CS NPs was conducted using transmission electron microscopy, Fourier-transform infrared spectroscopy, X-ray diffraction, and differential scanning calorimetry. Stability experiments, in vitro drug release studies, and lyophilisate selection were performed to evaluate the in vitro performance of HA@GRb1@CS NPs. The anti-inflammatory and antioxidant capabilities of HA@GRb1@CS NPs were assessed using H9c2 and RAW264.7 cells. Additionally, bioinformatics tools were employed to explore the mechanism of action of HA@GRb1@CS NPs in the treatment of CVDs associated with inflammation and oxidative stress. (3) Results: The optimal preparation process for HA@GRb1@CS NPs was achieved with a CS concentration of 2 mg/mL, a TPP concentration of 2.3 mg/mL, and a CS to TPP mass concentration ratio of 1.5:1, resulting in a particle size of 126.4 nm, a zeta potential of 36.8 mV, and a PDI of 0.243. Characterization studies confirmed successful encapsulation of the drug within the carrier, indicating successful preparation of HA@GRb1@CS NPs. In vitro evaluations demonstrated that HA@GRb1@CS NPs exhibited sustained-release effects, leading to reduced MDA (Malondialdehyde) content and increased SOD (Superoxide Dismutase) content in oxidatively damaged H9c2 cells. Furthermore, it showed enhanced DPPH (2,2-Diphenyl-1-picrylhydrazyl) and ABTS^+^ [2,2′-azino-bis(3-ethylbenzothiazoline-6-sulfonic acid)] free radical scavenging rates and inhibited the release of inflammatory factors NO (Nitric Oxide) and IL-6 (Interleukin-6) from RAW264.7 cells. (4) Conclusions: The HA@GRb1@CS NPs prepared in this study exhibit favorable properties with stable quality and significant anti-inflammatory and antioxidant capabilities. The mechanisms underlying their therapeutic effects on CVDs may involve targeting STAT3, JUN, EGFR, CASP3, and other pathways regulating cell apoptosis, autophagy, anti-lipid, and arterial sclerosis signaling pathways.

## 1. Introduction

Ginsenoside Rb1 (GRb1), an essential medicinal compound primarily extracted from the traditional Chinese herb ginseng, represents one of the major active constituents of ginseng and the most abundant monomeric ginsenoside [1]. It exhibits significant pharmacological effects, demonstrating promising efficacy in the treatment of CVDs [2,3], thus presenting itself as a highly potential active ingredient in traditional Chinese medicine for CVD therapy. The pharmacological actions of GRb1 mainly manifest in its remarkable anti-inflammatory and antioxidant capabilities [4], both of which are implicated as pathological factors in CVDs. Research findings [5,6] have indicated a close association between inflammation, oxidative stress, and the occurrence of heart failure, atherosclerosis, myocardial infarction, and hypertension.

Although GRb1 exhibits promising therapeutic effects against CVDs, its limited water solubility significantly restricts its bioavailability, thereby impeding its development and utilization. Nanotechnology is considered a multidisciplinary science that assists in solving current problems. It is defined as the control of matter at the atomic and molecular levels. Some fields where nanotechnology has been applied are the food industry. Within nanotechnology, nanoencapsulation is a strategy in recent years for the protection of compounds and improvements in the physicochemical and physiological properties of essential plant medicines to maintain or improve biological activity [7,8]. In previous studies [9,10], our team investigated the encapsulation of GRb1 into PLGA nanoparticles, demonstrating enhanced bioavailability and alleviating myocardial cell damage, consequently treating heart failure through the ROS/PPARα/PGC1α pathway. Building upon these findings, this study proposes a novel nanocarrier system—Hyaluronic Acid-Modified Ginsenoside Rb1 Self-Assembled Nanoparticles (HA@GRb1@CS NPs).

GRb1 is a saponin compound typically containing hydrophobic fatty acid moieties. In an aqueous environment, these hydrophobic groups may aggregate to form a hydrophobic core, serving as the initiation point for the self-assembly of GRb1 into nanoparticles [11,12]. Additionally, the hydroxyl and aromatic ring structures in GRb1 can promote mutual attraction and stacking between molecules through hydrogen bonding and π–π stacking interactions, facilitating the formation of stable self-assembled structures [13,14]. Hyaluronic acid (HA), a natural polysaccharide, possesses numerous hydroxyl and carboxyl groups in its molecular structure, providing active sites for receptor binding on cell surfaces, thus enhancing the targeting of GRb1 [15,16,17]. Chitosan (CS) exhibits excellent biocompatibility and biodegradability, posing no toxic side effects to the human body, and is recognized as a high-performance drug carrier [18]. Moreover, both HA and CS possess anti-inflammatory and antioxidant activities [19], which can synergize with GRb1 to effectively treat CVDs.

**Figure 1:**
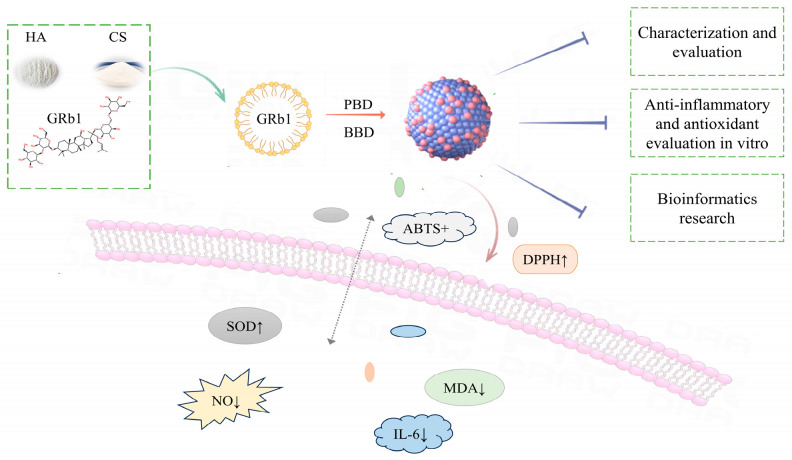
Schematic diagram of the HA@GRb1@CS NPs study.

Based on the above, this study aims to utilize HA modification and CS as a carrier to prepare HA@GRb1@CS NPs. The optimization of the preparation process is conducted to obtain a drug delivery system with appropriate particle size and high drug loading. Various characterization techniques are employed to validate the performance of HA@GRb1@CS NPs. Subsequently, the anti-inflammatory, antioxidant capabilities, and mechanism of action in treating CVDs of HA@GRb1@CS NPs are explored through in vitro cellular experiments and bioinformatics research; the schematic diagram of the study is shown in Figure 1.

## 2. Results

### 2.1. Results of Single-Factor Experiment

**Figure 2:**
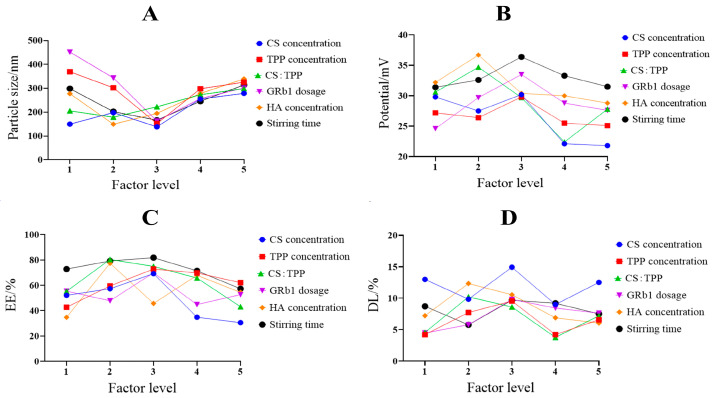
Results of single-factor experiment ((**A**) particle size, (**B**) potential, (**C**) EE, (**D**) DL).

The results of single-factor experiments are shown in Figure 2. The findings indicate that, when other factors are controlled, the particle size of HA@GRb1@CS NPs is minimized with a CS concentration of 2.5 mg/mL, a TPP concentration of 2 mg/mL, a CS to TPP mass concentration ratio of 2:1, a GRb1 dosage of 4 mg, an HA concentration of 0.2%, and a stirring time of 1.5 h, while the zeta potential, EE, and DL are maximized.

### 2.2. Significant Factors Identified through PBD

Based on the experimental design, 12 experiments were conducted, with each experiment repeated three times for averaging. The analysis of variance is presented in Table 1. The results show that the *p*-values for the models of particle size, EE, and DL are all less than 0.05, indicating that the established models are reliable. The findings indicate significant effects of CS concentration and TPP concentration on the particle size of HA@GRb1@CS NPs. Moreover, CS concentration and the mass ratio of CS to TPP significantly influence the EE of HA@GRb1@CS NPs, while CS concentration significantly affects the DL of HA@GRb1@CS NPs. Thus, three significant factors were identified through PBD–CS concentration, TPP concentration, and the mass ratio of CS to TPP.

### 2.3. BBD Optimizes the Preparation of HA@GRb1@CS NPs

**Figure 3:**
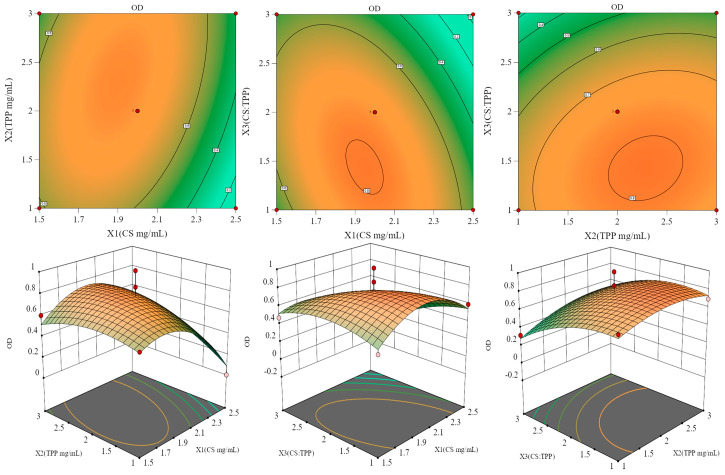
Three-dimensional response surface map of HA@GRb1@CS NPs preparation process.

The results of BBD are presented in Table 2 and Table 3. Regression analysis of experimental results was conducted using Design Expert 13.0 software, yielding a multivariate regression fitting equation: OD = −0.1362X_1_ + 0.0767X_2_ − 0.1491X_3_ + 0.1129X_1_X_2_ − 0.1476X_1_X_3_ + 0.0442X_2_X_3_ − 0.2556X_1_^2^ − 0.0907X_2_^2^ − 0.1192X_3_^2^ + 0.7588, with a R^2^ of 0.9467 and R_adj_^2^ of 0.7495. The model’s *p*-value is 0.0339, indicating significance (*p* < 0.05), while the lack-of-fit term has a *p*-value of 0.8265, suggesting insignificance, indicating a good fit. The coefficient of variation, CV%, is only 2.86%, indicating minimal error in the model equation, thus effectively reflecting experimental true values. To further elucidate the interaction among factors and optimize the preparation process, response surface visualization analysis was conducted. The three-dimensional response surface plots are shown in Figure 3. Among them, the response surface plot of X_1_ and X_2_ is the steepest, indicating the most significant interaction between CS concentration and TPP concentration. Based on the response surface results, the optimal preparation process is determined: CS concentration of 2 mg/mL, TPP concentration of 2.3 mg/mL, and a CS to TPP mass concentration ratio of 1.5:1.

### 2.4. Characterization of HA@GRb1@CS NPs

**Figure 4:**
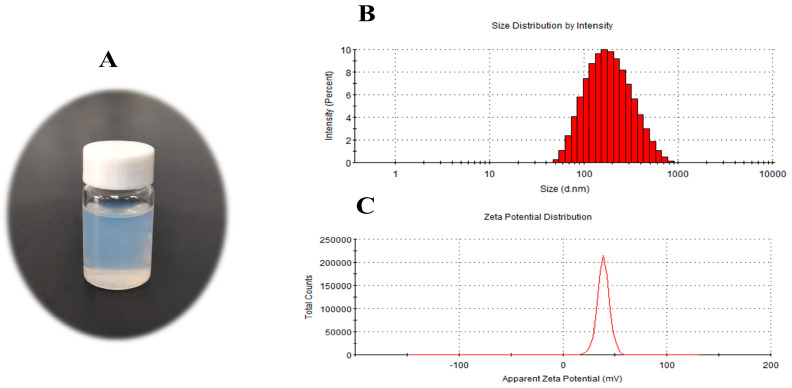
(**A**) Visual representation of HA@GRb1@CS NPs, (**B**) particle size, and (**C**) potential.

The HA@GRb1@CS NPs prepared under the fabrication process are colloidal suspensions exhibiting a faint blueish micro-bright appearance, characterized by clarity, transparency, and absence of impurities or precipitates. Their particle size was determined to be 126.4 nm, with a surface potential of 36.8 mV and a PDI of 0.243, as shown in Figure 4.

#### 2.4.1. Morphological Observation

**Figure 5:**
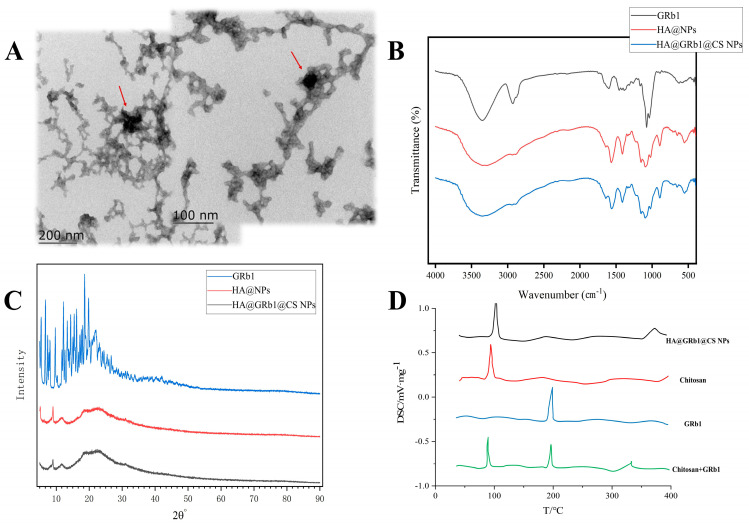
Characterization of HA@GRb1@CS NPs, (**A**) TEM image (the arrow indicates GRb1), (**B**) FTIR spectrum, (**C**) XRD spectrum, (**D**) DSC spectrum.

TEM images reveal the well-organized appearance of HA@GRb1@CS NPs, indicating successful preparation with GRb1 anchored onto the carrier. See TEM image in Figure 5A for details.

#### 2.4.2. FTIR Analysis

FTIR analysis revealed a characteristic band of GRb1 at around 3000 cm^−1^ attributed to -C-H- stretching vibrations and at 1672 cm^−1^ indicating -N-H- bending vibrations, confirming the presence of hydrocarbon and amino functional groups in GRb1. The absorption band at these positions notably weakened or disappeared in HA@GRb1@CS NPs, suggesting successful encapsulation. In HA@NPs, characteristic bands of CS were predominant, with a band at 1725 cm^−1^ attributed to -C=O- stretching vibrations of CS aldehyde groups and at 1178 cm^−1^ corresponding to -C-O- stretching vibrations of sugar rings. Comparable band observed in HA@GRb1@CS NPs at identical positions, as depicted in Figure 5B.

#### 2.4.3. XRD Analysis

The XRD pattern reveals distinct characteristic peaks of GRb1, while no obvious peaks of GRb1 are observed in the XRD pattern of HA@GRb1@CS NPs. The overall spectrum resembles that of HA@NPs, indicating successful encapsulation of GRb1 by CS and the preservation of nanomaterial properties intact, as shown in Figure 5C.

#### 2.4.4. DSC Analysis

The DSC results are shown in Figure 5D. The characteristic endothermic peak of drug GRb1 appears at 196 °C, while CS exhibits a peak at 80 °C, consistent with literature-reported melting points of GRb1 and CS. The endothermic peaks of the drug and carrier appear at the corresponding positions in the CS and GRb1 mixture. However, HA@GRb1@CS NPs only show the melting endothermic peak of CS without the presence of the drug’s endothermic peak, indicating that GRb1 exists in an amorphous state within the HA@GRb1@CS NPs, suggesting successful encapsulation of the drug after formation into nanoparticles.

### 2.5. Results of Stability Study

**Figure 6:**
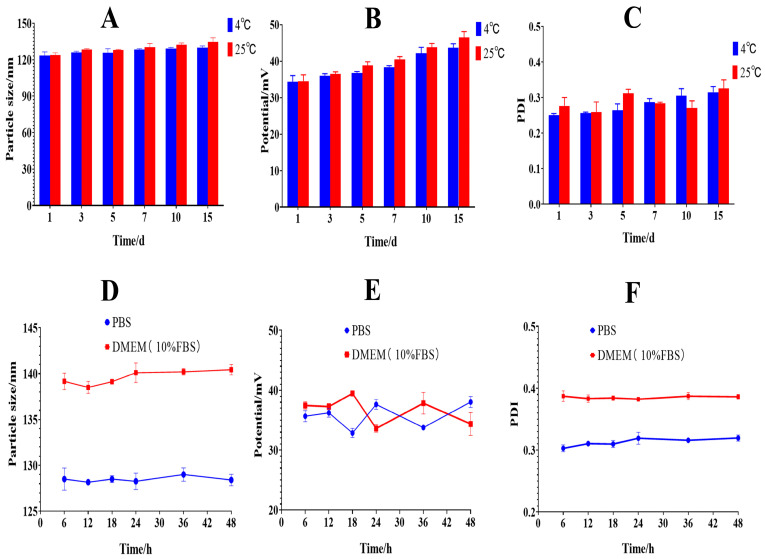
Stability Study of HA@GRb1@CS NPs ((**A**–**C**) are particle size, potential, and PDI changes of HA@GRb1@CS NPs at 4 °C and 25 °C, *n* = 3. (**D**–**F**) are particle size, potential, and PDI changes of HA@GRb1@CS NPs in PBS, DMEM, *n* = 3).

To assess the storage stability of HA@GRb1@CS NPs, they were kept at 4 °C and 25 °C. Results indicate that within 15 d, there were no significant changes in particle size and PDI at both temperatures. However, over time, there was a slight increase in potential, likely attributed to CS’s characteristics as a polyelectrolyte, which can adsorb surrounding ions and induce aggregation, resulting in an increase in positive charge. The various indicators of HA@GRb1@CS NPs show no significant difference when stored at 25 °C and 4 °C. To avoid potential microbial contamination at 25 °C, we chose to store them at 4 °C. To investigate the stability of HA@GRb1@CS NPs in different media, PBS and DMEM containing 10% FBS were selected as dialysis media for the stability experiments. Results reveal minimal changes in particle size and PDI in both media, while potential exhibited fluctuations, possibly due to dynamic changes in the ionic environment. Compared with PBS, HA@GRb1@CS NPs showed increased particle size and PDI in DMEM, attributed to adsorption of proteins and other biomolecules present in DMEM. Stability assessments under different temperatures and media are depicted in Figure 6.

### 2.6. Results of In Vitro Drug Release

**Figure 7:**
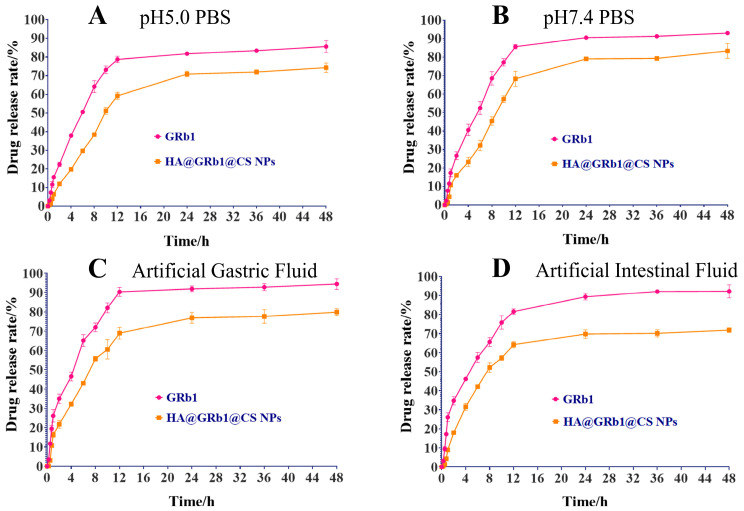
Drug release profiles of HA@GRb1@CS NPs in different media ((**A**) pH = 5.0 PBS, (**B**) pH = 7.4 PBS, (**C**) artificial gastric fluid, (**D**) artificial intestinal fluid, *n* = 3).

The drug release profiles of HA@GRb1@CS NPs and GRb1 in different media were plotted with drug dialysis time as the abscissa and the cumulative release percentage of GRb1 as the ordinate, as shown in Figure 7. It is evident from the graph that, compared with GRb1, HA@GRb1@CS NPs exhibit a significantly slower drug release rate, indicating a sustained-release effect. Within 48 h, the cumulative drug release rate of GRb1 ranged from 85% to 94%, whereas that of HA@GRb1@CS NPs ranged from 71% to 83%, suggesting that converting GRb1 into nanoparticles can prolong its release duration. Model fitting revealed that the drug release of HA@GRb1@CS NPs in the four media conforms to a first-order kinetic model, as shown in Table 4.

### 2.7. Lyophilization Protection Technology Study

**Figure 8:**
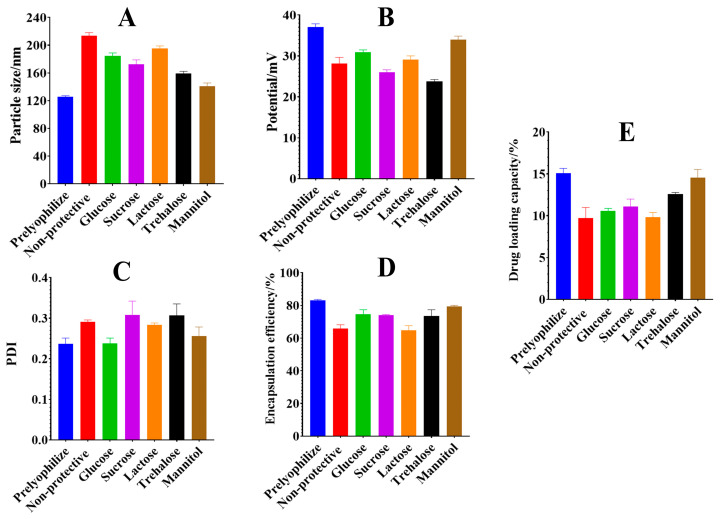
Effect of different lyoprotectants on HA@GRb1@CS NPs ((**A**) particle size, (**B**) potential, (**C**) PDI, (**D**) EE, (**E**) DL, n = 3).

Upon comprehensive evaluation of various indicators of HA@GRb1@CS NPs, results indicate no significant changes in particle size and zeta potential when mannitol is incorporated as a lyoprotectant, as depicted in Figure 8. Moreover, the nanocarrier exhibits a soft texture post-lyophilization, with no residue upon reconstitution with pure water. Compared with other protectants, mannitol exerts the least impact on HA@GRb1@CS NPs, hence being selected as the most suitable lyoprotectant. To determine the optimal concentration of mannitol, we examined lyoprotective effects at mass fractions of 1%, 2%, 5%, 10%, and 20% mannitol. As shown in Table 5, turbidity and insoluble residues appear upon reconstitution of HA@GRb1@CS NPs when the mannitol mass fraction exceeds 5%. Compared with 5% mannitol, 1% and 2% mannitol had a greater impact on particle size after lyophilization. Therefore, in this study, we opt for 5% mannitol for lyoprotecting HA@GRb1@CS NPs.

### 2.8. In Vitro Cytotoxicity of HA@GRb1@CS NPs

**Figure 9:**
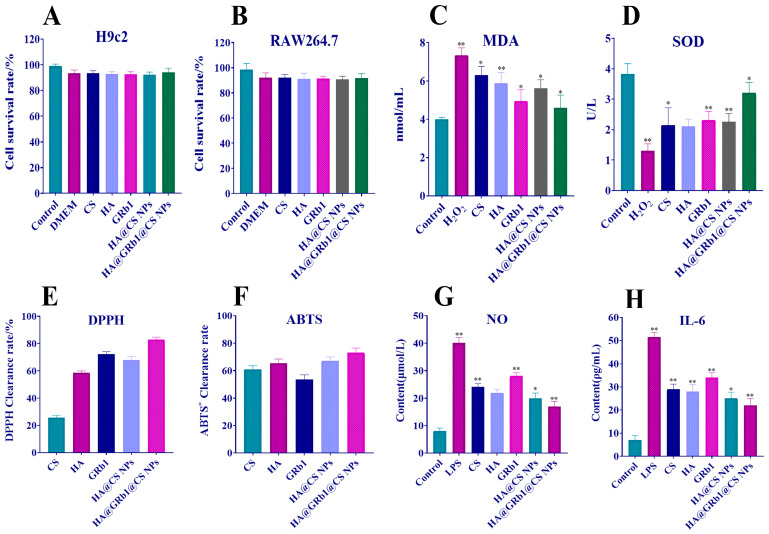
(**A**) H9c2 cell cytotoxicity assay, (**B**) RAW264.7 cell cytotoxicity assay, (**C**) Changes in MDA content of H9c2 cells, (**D**) Changes in SOD content of H9c2 cells, (**E**) DPPH free radical scavenging rate, (**F**) ABTS^+^ free radical scavenging rate, (**G**) Changes in NO content of RAW264.7 cells, (**H**) Changes in IL-6 content of RAW264.7 cells, n = 6, * *p* < 0.05 and ** *p* < 0.01 vs. Control group.

This study assessed the toxicity of HA@GRb1@CS NPs using H9c2 and RAW264.7 cell lines. The results showed that HA@GRb1@CS NPs exhibited no obvious toxicity to both cell types. Neither the carrier CS nor the modifier HA noticeably affected cell viability, indicating the safety and biocompatibility of the materials used in HA@GRb1@CS NPs. Cell viability results are shown in Figure 9A,B.

### 2.9. HA@GRb1@CS NPs Reduces MDA Content and Elevates SOD Content in Oxidatively Damaged H9c2 Cells

Compared with the control group, the intracellular MDA content of H9c2 cells significantly increased, and the SOD content significantly decreased after oxidative damage induced by H_2_O_2_. After drug treatment, it can be clearly observed that oxidative damage is alleviated. HA@GRb1@CS NPs can reduce the intracellular MDA content and increase SOD content in H9c2 cells, as shown in Figure 9C,D. Moreover, compared with GRb1 treatment alone, HA@GRb1@CS NPs demonstrate superior efficacy. CS and HA also exhibit certain antioxidant effects.

### 2.10. HA@GRb1@CS NPs Improves DPPH, ABTS^+^ Radical SCAVENGING

As shown in Figure 9E,F, CS, HA, GRb1, and HA@GRb1@CS NPs all exhibit antioxidant activity, but there are no significant differences among the groups. Compared with GRb1, HA@GRb1@CS NPs can enhance the DPPH and ABTS^+^ radical scavenging rates, reaching 82.7% and 74.6%, respectively. This enhancement may be attributed to the improved stability of GRb1 when formulated into nanoparticles, thereby prolonging its pharmacological effects. Additionally, the synergistic effect of CS and HA may also contribute to the enhanced antioxidant activity.

### 2.11. HA@GRb1@CS NPs Inhibit RAW264.7 Cells Inflammatory Factor Release of NO, IL-6

Following LPS induction, RAW264.7 cells release a significant amount of NO and IL-6, triggering inflammatory responses. Compared with the model group, four substances, namely CS, HA, GRb1, and HA@GRb1@CS NPs, alleviate the inflammatory response of RAW264.7 cells to varying degrees, suppressing the release of NO and IL-6. Among these, GRb1 exhibits the weakest anti-inflammatory capability, while HA@GRb1@CS NPs demonstrate the strongest. This suggests that the incorporation of GRb1 into nanoparticles, with the support of CS and HA, significantly enhances its anti-inflammatory properties, as depicted in Figure 9G,H.

### 2.12. Bioinformatics Analysis Results of HA@GRb1@CS NPs in the Treatment of CVDs

#### 2.12.1. Differential Gene Expression in CVDs

**Figure 10:**
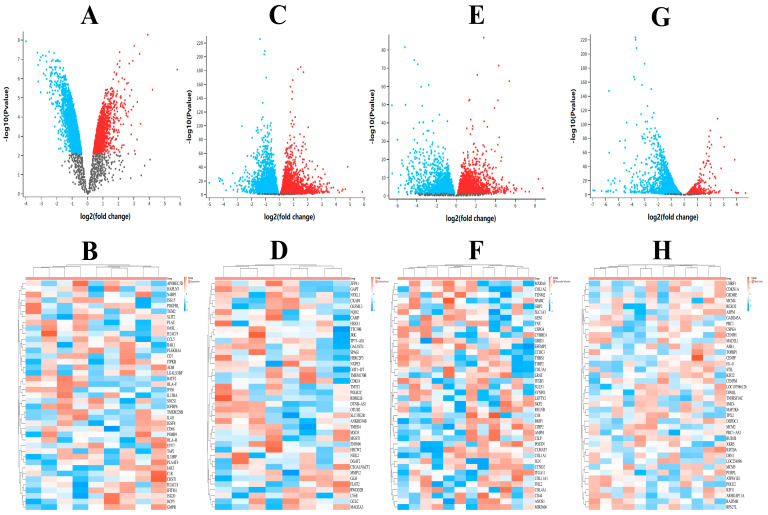
Differential gene volcano and heat map analysis of CVDs ((**A**,**B**) heart failure, (**C**,**D**) atherosclerosis, (**E**,**F**) myocardial infarction, (**G**,**H**) hypertension).

Using the GEO2R function, we analyzed twelve samples of heart failure and identified 1578 differentially expressed genes; eight samples of atherosclerosis, resulting in 983 differentially expressed genes; twelve samples of myocardial infarction, yielding 1271 differentially expressed genes; and nine samples of hypertension, revealing 1104 differentially expressed genes. As shown in Figure 10, the volcano plot depicts upregulated genes in red, downregulated genes in blue, and genes with insignificant differential expression in gray. The heatmap indicates gene expression levels, with red representing upregulation and blue representing downregulation, suggesting differential gene expression profiles among the samples.

#### 2.12.2. Target Screening for Intersection of HA@GRb1@CS NPs and CVDs

**Figure 11:**
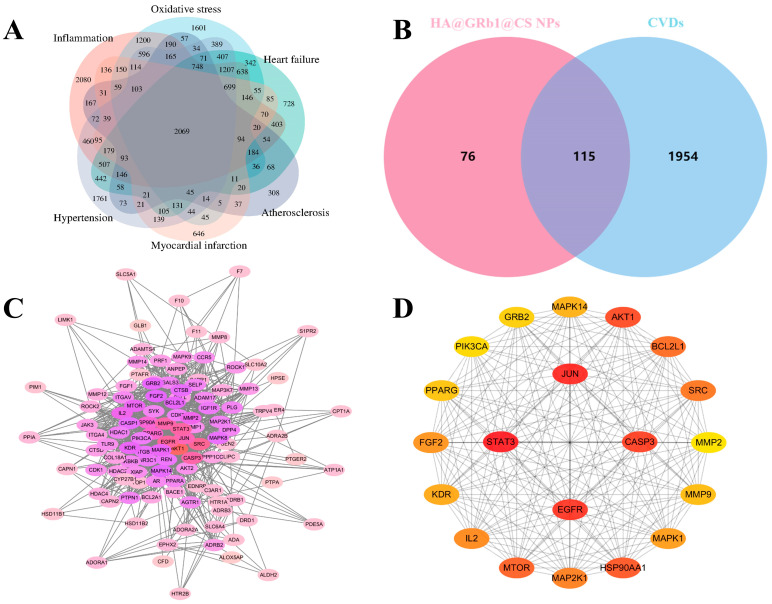
(**A**) Wayne’s map of targets of CVDs associated with inflammation and oxidative stress; (**B**) Wayne’s map of intersecting targets of HA@GRb1@CS NPs and CVDs; (**C**) PPI map; (**D**) Hub gene map.

Through screening, a total of 191 targets were identified for HA@GRb1@CS NPs, while 2069 targets associated with inflammation and oxidative stress-related CVDs were obtained. By utilizing the Venny online platform for matching, a final intersection of 115 targets between HA@GRb1@CS NPs and CVDs was obtained, which represents the therapeutic targets of HA@GRb1@CS NPs for treating CVDs, as depicted in the Venn diagram shown in Figure 11A,B.

#### 2.12.3. PPI Network Construction

Importing intersecting targets from the STRING database, the network diagram contains 115 nodes and 1140 edges, with an average degree value of 19.8. The PPI network diagram is shown in Figure 11C. Through core gene screening, the top four targets ranked by degree are STAT3, JUN, EGFR, and CASP3. The hub gene diagram is shown in Figure 11D.

#### 2.12.4. GO and KEGG Analysis

**Figure 12:**
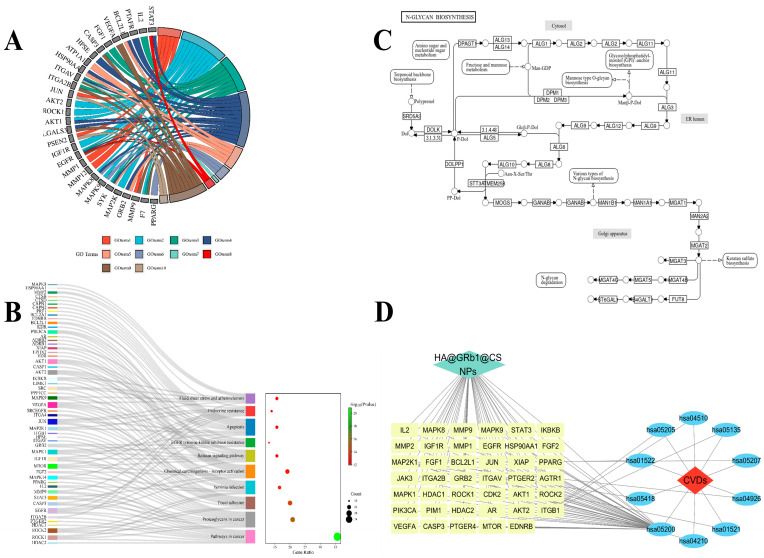
(**A**) GO analysis, (**B**) KEGG analysis, (**C**) KEGG mechanism of action map, (**D**) topological network map.

The GO analysis yielded 657 biological process (BP) entries, 134 cellular component (CC) entries, and 108 molecular function (MF) entries, respectively, with the top 10 rankings depicted in the chord diagram shown in Figure 12A. GO enrichment results indicated that HA@GRb1@CS NPs therapy for CVDs primarily involves biological processes such as long-chain fatty acid biosynthesis and organism inflammation, cellular components including endoplasmic reticulum and cytoplasmic membrane, and molecular functions such as activation of protein kinases and oxidoreductase activity. KEGG analysis identified 149 pathways, with the top 10 depicted in the bubble chart shown in Figure 12B. The results suggest that the mechanism of action of HA@GRb1@CS NPs in treating CVDs may be associated with signaling pathways involving cell apoptosis, autophagy, anti-lipid, and arterial sclerosis, as illustrated in Figure 12C.

#### 2.12.5. Drug-Target Pathway Disease Topological Network Analysis

A visualization of network topology analysis was conducted using the Network Analyzer plugin, revealing a total of 41 target nodes implicated in the shared interaction between drugs and pathways. This signifies the therapeutic impact of HA@GRb1@CS NPs on treating CVDs through these 41 key targets, as depicted in Figure 12D. In the established topological network graph, drug HA@GRb1@CS NPs are denoted in green, targets in yellow, pathways in blue, and CVDs in red.

#### 2.12.6. Molecular Docking Validation Results

**Figure 13:**
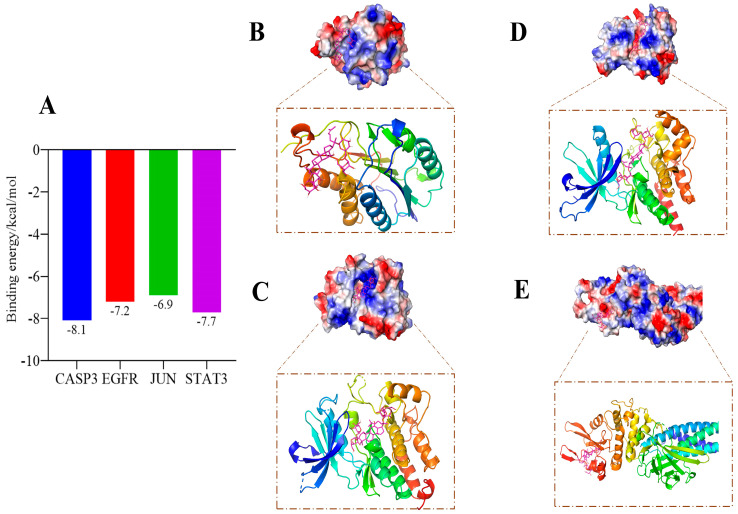
(**A**) Molecular docking binding energy results; (**B**–**E**) GRb1 and STAT3, JUN, EGFR, CASP3 target visualization results.

The binding affinities between GRb1 and target proteins STAT3, JUN, EGFR, and CASP3 are shown in Figure 13A. The docking binding energies are all less than −6 kcal/mol, indicating a strong binding interaction and high compatibility between GRb1 and each target protein. Molecular docking visualization analysis reveals that GRb1 exhibits favorable binding modes with STAT3, JUN, EGFR, and CASP3, forming stable complexes with these proteins and demonstrating strong correlations with the targets, as depicted in Figure 13B–E.

## 3. Discussion

Inflammation and oxidative stress are pivotal factors in the occurrence and progression of CVDs, closely associated with conditions such as heart failure, atherosclerosis, myocardial infarction, and hypertension [20,21,22,23]. Inflammatory responses can lead to endothelial damage, with inflammatory cells such as macrophages and T lymphocytes accumulating in the vessel wall, releasing inflammatory mediators such as interleukins and tumor necrosis factor [24]. This process promotes endothelial cell proliferation and lipid deposition, forming plaques, ultimately resulting in vessel narrowing and plaque rupture, precipitating myocardial infarction [25]. Additionally, oxidative stress can directly impair endothelial cells, disrupting their normal function, promoting vascular inflammation by increasing the oxidation of low-density lipoprotein cholesterol, enhancing its damaging effects on the vessel wall, and accelerating the progression of atherosclerosis [26]. Furthermore, inflammation and oxidative stress can cause myocardial cell damage and fibrosis, affecting cardiac contraction and relaxation function and exacerbating the severity of heart failure [27]. Therefore, inhibiting the occurrence of inflammatory reactions and oxidative stress is crucial for treating CVDs.

GRb1 exhibits therapeutic potential in treating CVDs. Studies have shown [28] that GRb1 promotes proteasome degradation, exerting a protective effect in mouse models of acute myocardial ischemia and oxygen–glucose deprivation-induced myocardial cell damage. Results indicate that GRb1 significantly enhances AMPKα phosphorylation, promoting mitochondrial autophagy, thereby reducing myocardial infarction size and alleviating myocardial damage. In a mouse model of atherosclerosis induced by a high-fat diet [29], oral administration of GRb1 was found to suppress levels of inflammatory factors such as IL-1β, IL-6, and TNF-α in serum, attenuating apoptosis associated with inflammatory activity. Further research [30] has confirmed that GRb1 mediates cardiac protection by modulating various signaling pathways, including mitochondrial apoptotic pathways, PI3K/Akt/mTOR, HIF-1α and GRF91, RhoA, and p38α MAPK, while also improving biochemical parameters of oxidative stress and inflammation.

To enhance the therapeutic efficacy of GRb1 against CVDs, we formulated it into nanoparticles. Nanoparticles represent a widely employed nanoformulation for drug delivery, offering dual benefits. Firstly, they encapsulate drugs, forming stable drug carriers that shield drugs from enzymatic, acidic, and immune degradation, thereby prolonging drug half-life and enhancing bioavailability [31,32,33]. Secondly, they facilitate efficient drug delivery to tissues or cells via bloodstream and lymphatic systems, traversing biological barriers more effectively [34,35]. Additionally, HA modification confers greater targeting ability to GRb1. HA binds to CD44 receptors overexpressed on the surfaces of many tumor and inflammatory cells [36]. HA modification facilitates CD44 receptor recognition and absorption, thereby increasing drug accumulation within target tissues or cells while minimizing damage to healthy tissues [37]. Cell experiments corroborate the enhanced anti-inflammatory and antioxidant activities of HA@GRb1@CS NPs compared with GRb1 alone, suggesting a synergistic effect of HA and CS.

The “Treatment of Different Diseases with the Same Method” is a common practice in traditional Chinese medicine, which involves analyzing the root cause of diseases and applying similar therapeutic approaches to various ailments. Given that inflammation and oxidative stress can both affect the onset of cardiovascular diseases such as heart failure, atherosclerosis, myocardial infarction, and hypertension, treatments targeting inflammation and oxidative stress perspectives, such as HA@GRb1@CS NPs, may effectively address these cardiovascular conditions. Bioinformatics research indicates a close correlation between the targets of inflammation, oxidative stress, and CVDs. Gene enrichment analysis suggests that HA@GRb1@CS NPs may potentially treat CVDs through core targets like STAT3, JUN, EGFR, and CASP3. Both STAT3 and JUN are common targets in inflammatory responses. Studies have shown that JUN is closely related to the JAK2/STAT3 signaling pathway and the PI3K-Akt signaling pathway, and it can effectively treat acute renal injury caused by myocardial ischemia [38]. EGFR, a type of epidermal growth factor receptor, has been extensively studied in the field of CVDs. Research indicates that [39] inhibiting the EGFR/IGF1R signaling pathway can significantly improve and restore myocardial relaxation and contraction function. CASP3 is a key protease that affects cell apoptosis, and studies suggest it as a potential target for heart failure. After inducing injury to H9c2 cells with H_2_O_2_, CASP3 levels significantly increase, but this increase is inhibited after drug treatment [40]. Therefore, the therapeutic effect of HA@GRb1@CS NPs on CVDs is likely associated with the regulation of these aforementioned targets.

## 4. Materials and Methods

### 4.1. Materials

Ginsenoside Rb1 was purchased from Chengdu Alpha Biological Technology Co., Ltd. (Chengdu, China). Hyaluronic acid, chitosan, and sodium tripolyphosphate were purchased from Shanghai Macklin Biochemical Technology Co., Ltd. (Shanghai, China). Acetic acid, acetonitrile, and phosphoric acid were purchased from China National Pharmaceutical Group Corporation (Nanjing, China). CCK-8, fetal bovine serum, and PBS were purchased from Beijing Boaosen Biotechnology Co., Ltd. (Beijing, China). SOD, MDA, DPPH, and ABTS^+^ assay kits were purchased from Shanghai Beyotime Biotechnology Co., Ltd. (Shanghai, China). IL-6 and NO assay kits were purchased from Wuhan Beyotime Biotechnology Co., Ltd. (Wuhan, China). Lipopolysaccharide (LPS) was purchased from Sigma-Aldrich (Chengdu, China). RAW264.7 and H9c2 cell lines were obtained from the Cell Bank of the Chinese Academy of Sciences (Shanghai, China) and cultured at 37 °C with 5% CO_2_.

### 4.2. Preparation of HA@GRb1@CS NPs

HA@GRb1@CS NPs were prepared by the ionic gel method [41]: 20 mg of CS was weighed and dissolved in 10 mL of water ultrasonically, stirred at 1200 rpm with a magnetic stirrer (Aika Instrument Equipment Co., Ltd., Guangzhou, China), and 200 μL of acetic acid was added slowly to promote the dissolution of CS. Separately, 3 mg of GRb1 and 10 mg of sodium tripolyphosphate (TPP) were weighed and dissolved in 10 mL of water using ultrasonic treatment. Additionally, 1 mg of HA was weighed and dissolved in 1 mL of water. Using a syringe, the GRb1-TPP solution and HA solution were carefully added to the CS solution under the liquid surface. The mixture was stirred at 600 rpm for 1 h, followed by ultrasonic crushing for 20 min, and then passed through 0.22 μm filter membrane to obtain HA@GRb1@CS NPs.

### 4.3. Determination of Particle Size, Potential, Encapsulation Efficiency (EE), and Drug Loading Capacity (DL)

An amount of 2 mL of nanoparticle suspension was taken and subjected to Malvern particle size analyzer to measure the particle size and zeta potential of HA@GRb1@CS NPs. EE and DL were determined by HPLC using a Hyperdil BDS C18 column (Thermo Fisher Scientific Co., Ltd., Shanghai, China, 4.6 mm × 200 mm, 5 µm), with a detection wavelength of 203 nm and a mobile phase consisting of acetonitrile and 0.05% phosphoric acid. The gradient elution was as follows: 0–10 min, 35–40% acetonitrile; 10–15 min, 35–40% acetonitrile; flow rate was set at 1.2 mL/min, column temperature at 30 °C, and injection volume at 10 μL. HA@GRb1@CS NPs were centrifuged at 12,000 rpm for 30 min to collect the supernatant for HPLC analysis of the free drug content. EE% = (Wtotal − Wdissociate)/Wtotal × 100%, where Wtotal represents the total drug weight and Wdissociate represents the weight of dissociated drug. Additionally, 1 mL of HA@GRb1@CS NPs was precisely transferred without adding any lyoprotectant and lyophilized for 48 h. The lyophilized powder was weighed, dissolved in methanol, and subjected to HPLC to determine the total GRb1 content, DL% = Wtotal/Wlyophilized × 100%, where Wtotal represents the total drug weight and Wlyophilized represents the weight of the lyophilized powder.

### 4.4. Single-Factor Experiment

Preparation of HA@GRb1@CS NPs, single-factor experiments were conducted to investigate the effects of six factors, including CS concentration (1.5, 2, 2.5, 3, 3.5 mg/mL), TPP concentration (0.5, 1, 2, 3, 4 mg/mL), CS to TPP mass concentration ratio (1:1, 2:1, 3:1, 4:1, 5:1), GRb1 dosage (2, 3, 4, 5, 6 mg), HA concentration (0.1, 0.2, 0.3, 0.4, 0.5%), and stirring time (0.5, 1, 1.5, 2, 2.5 h), on the particle size, zeta potential, EE, and DL of HA@GRb1@CS NPs.

### 4.5. Plackett–Burman Design (PBD)

Performing PBD experiments to screen significant factors affecting the preparation of HA@GRb1@CS NPs involves multiple parameters, including CS concentration (A), TPP concentration (B), the mass concentration ratio of CS to TPP (C), GRb1 dosage (D), HA concentration (E), and stirring time (F). Based on the optimal levels obtained from single-factor experiments, experiments are designed using the high (+1) and low (−1) levels before and after the optimal levels. Particle size, zeta potential, EE, and DL are evaluated as criteria, and Design-Expert 13.0 software is utilized for experimental design. Specific factors and levels are detailed in Table 6.

### 4.6. Box–Behnken Design (BBD)

Using the significant factors screened by PBD as independent variables: CS concentration (X_1_), TPP concentration (X_2_), and the mass concentration ratio of CS to TPP (X_3_), each factor is set at three levels: high (+1), medium (0), and low (−1). A three-factor, three-level experimental design was conducted as shown in Table 7, employing BBD optimization for the preparation process of HA@GRb1@CS NPs, with particle size, zeta potential, EE, and DL as response values. Due to the abundance of response values, each response was normalized to a range of 0 to 1, and the geometric mean was calculated to obtain an overall normalized value (OD). OD = (d_1_d_2_…d_k_)^1/k^, where k represents the number of response values. Hassan’s method was utilized to calculate the normalized values, dmax and dmin, for factors where lower values are desirable and factors where higher values are desirable. dmax = (Yi − Ymin)/(Ymax − Ymin), dmin = (Ymax − Yi)/(Ymax − Ymin).

### 4.7. Characterization of HA@GRb1@CS NPs

#### 4.7.1. Transmission Electron Microscopy (TEM) Morphological Observations

Preparation of HA@GRb1@CS NPs followed a specific protocol: 1 mL of HA@GRb1@CS NPs was diluted with 4 mL of distilled water. Subsequently, 10 μL of the diluted HA@GRb1@CS NPs was dropped onto a copper grid, followed by 30 min of incubation. Afterward, staining was performed using 1% phosphotungstic acid, excess liquid was removed, and observations were conducted under TEM (Beijing, China).

#### 4.7.2. Fourier Transform Infrared Spectroscopy (FTIR) Analysis

The structure and composition of HA@GRb1@CS NPs were characterized using FTIR technique. A total of 20 mg each of lyophilized HA@GRb1@CS NPs, GRb1, and HA@NPs was thoroughly mixed with an appropriate amount of dried potassium bromide powder, followed by pelletization. The pellets were then placed in the sample window of Nicolet iS 10 (Thermo Fisher Scientific Co., LTD, Shanghai, China) for detection. Infrared spectra absorption peaks of the samples were collected in the wavenumber range of 400–4000 cm^−1^.

#### 4.7.3. X-ray Diffraction (XRD) Analysis

A total of 15 mg each of freeze-dried HA@GRb1@CS NPs, GRb1, and HA@NPs was taken. After the XRD instrument (Guangzhou Beituo Science and Technology Co., Ltd., Guangzhou, China) stabilized, the samples were placed on the sample holder for structural characterization. The scanning range was set from 5 to 90° with a diffraction angle of 2°. Diffraction peaks of the samples were collected, and a diffraction pattern was plotted.

#### 4.7.4. Differential Scanning (DSC) Analysis

A total of 20 mg of freeze-dried HA@GRb1@CS NPs, GRb1, CS, and their mixture were placed in crucibles. The samples were tested under a nitrogen atmosphere with a heating rate of 10 °C/min from 30 to 400 °C. Thermal enthalpy data were collected, and thermal analysis curves were plotted accordingly.

### 4.8. Stability Study

HA@GRb1@CS NPs were prepared according to the synthesis protocol and subsequently stored at 4 °C and 25 °C for 1, 3, 5, 7, 10, and 15 d, respectively. Additionally, to mimic the in vivo conditions, HA@GRb1@CS NPs were mixed with PBS and DMEM culture medium (containing 10% fetal bovine serum) and incubated at 37 °C with agitation (200 rpm) for 6, 12, 18, 24, 36, and 48 h. Samples were taken at different time points under various temperature and medium conditions to assess changes in particle size, zeta potential, and PDI.

### 4.9. Drug Release Studies In Vitro

The dynamic dialysis method [42] was employed to assess the in vitro drug release profile of HA@GRb1@CS NPs. Boiled dialysis bags were prepared and filled with 3 mL of HA@GRb1@CS NPs and GRb1. These bags were immersed in PBS at pH 5.0, PBS at pH 7.4, simulated gastric fluid (prepared in-house in the laboratory), and simulated intestinal fluid (prepared in-house in the laboratory) and subjected to dialysis at 200 rpm. Samples (1 mL) were withdrawn at predetermined time points (0, 0.25, 0.5, 0.75, 1, 2, 4, 6, 8, 12, 24, and 48 h), with an equivalent volume of fresh medium added after each withdrawal. GRb1 content in the samples was determined by HPLC after membrane filtration, cumulative drug release was calculated, and drug release kinetics were modeled.

### 4.10. Lyophilization Protection Studies

To prepare HA@GRb1@CS NPs, 2 mL of the suspension was drawn into a vial and mixed with different types of lyoprotectants (glucose, sucrose, lactose, trehalose, mannitol; mass fractions are all 1%). After freeze-drying the mixture for 48 h, the original volume of pure water was added to re-dissolve it, and changes in particle size, zeta potential, PDI, EE, and DL were measured to determine the optimal cryoprotectant. Subsequently, the concentration of the selected lyoprotectant was further investigated and validated.

### 4.11. In Vitro Evaluation of Anti-Inflammatory and Antioxidant Activities

#### 4.11.1. Cytotoxicity Assay

RAW264.7 and H9c2 cells were seeded in 96-well plates and cultured for 24 h. They were then divided into control, DMEM, CS, HA, GRb1, and HA@GRb1@CS NPs groups (40 μg/mL). After incubating with respective drugs in the incubator for 24 h, cell viability was assessed using the CCK-8 assay. Each well was added with 100 μL of 10% CCK-8 reagent and incubated at 37 °C for 1.5 h until the color turned orange. Absorbance OD values were measured at 450 nm using a microplate reader. Cell viability = (OD sample group − OD control group)/OD control group.

#### 4.11.2. Evaluation of HA@GRb1@CS NPs against Oxidative Damage in H9c2 Cells

An oxidative damage model was established in H9c2 cardiomyocytes by treating with 400 μM H_2_O_2_ for 4 h. Following the modeling, cells were treated with CS, HA, GRb1, or HA@GRb1@CS NPs (40 μg/mL) for 12 h according to grouping. The supernatant was collected for analysis of SOD and MDA levels using assay kits.

#### 4.11.3. Determination of DPPH, ABTS^+^ Radical Scavenging by HA@GRb1@CS NPs

CS, HA, GRb1, and HA@GRb1@CS NPs were individually prepared into solutions with a concentration of 40 μg/mL. Then, 100 μL of each solution was mixed with an equal volume of DPPH solution and kept in the dark for 30 min. The absorbance was measured at 515 nm (A1). Similarly, the absorbance values were determined for the sample control group by mixing the solutions with an equal volume of DMEM (A2) and for the blank control group by mixing DMEM with DPPH solution (A0). DPPH scavenging rate = (A0 − A1 + A2)/A0 × 100%. Additionally, 10 μL of the above solution was added to 100 μL of ABTS^+^ working solution and allowed to react in the dark for 7 min. Absorbance was measured at 734 nm (A1). Another 10 μL of DMEM was reacted with 100 μL of ABTS^+^ to determine the absorbance value (A0) as a blank control. ABTS^+^ scavenging rate = (A0 − A1)/A0 × 100%.

#### 4.11.4. Evaluation of HA@GRb1@CS NPs in Anti-Inflammatory Activity in RAW264.7 Cells

An inflammatory model of RAW264.7 cells was established by inducing with 1 μg/mL LPS. After model establishment, cells were treated with CS, HA, GRb1, or HA@GRb1@CS NPs (40 μg/mL) for 24 h according to grouping. The levels of inflammatory factors IL-6 and NO in the cell supernatant were measured using ELISA and Griess reagent kits, respectively, following the manufacturer’s instructions.

### 4.12. Bioinformatics Analysis of HA@GRb1@CS NPs for the Treatment of CVDs

#### 4.12.1. Differential Gene Acquisition in CVDs

Using “heart failure”, “atherosclerosis”, “myocardial infarction”, and “hypertension” as keywords, gene expression profiles (GSE231597, GSE247722, GSM789924, GSM7473722) were downloaded from the GEO database. Differential expression genes in various CVDs were identified by setting criteria of *p* < 0.05 and |log FC| ≥ 2, and visualized through volcano plots. The top 40 genes were further selected and represented in a heatmap.

#### 4.12.2. HA@GRb1@CS NPs and CVDs Intersection Target Acquisition

The GRb1-related targets were obtained from the TCMSP database, and the SMILES format of GRb1 was downloaded from the PubChem database. These were then imported into the Swiss Target Prediction database to obtain target predictions for the constituents. Following the principle of “Treatment of Different Diseases with the Same Method”, relevant targets were obtained using keywords such as “inflammation”, “oxidative stress”, “heart failure”, “atherosclerosis”, “myocardial infarction”, and “hypertension”. The common targets were selected as disease targets for CVDs caused by inflammation and oxidative stress. Subsequently, the targets were imported into the Venny2.1.0 online platform for intersection matching and Venn diagram plotting.

#### 4.12.3. Protein Interaction Network (PPI) Construction

Import the intersection targets into the STRING database with organism type set as “Homo sapiens”. Import the TSV format file into Cytoscape 3.8.2 software and visualize the PPI network. Then, filter the top 20 targets based on their degree values to generate a Hub gene diagram.

#### 4.12.4. Functional (GO) and Pathway Enrichment (KEGG) Analyses

GO and KEGG analyses were conducted using Metascape and David databases, with species limited to “Homo sapiens”, applying thresholds of *p* < 0.05 and FDR < 0.05 for selection. Chord diagrams were generated for the top 10 ranked GO terms, while bubble and pathway diagrams were created for the top 10 ranked KEGG pathways, illustrating their functional mechanisms.

#### 4.12.5. Drug-Target Pathway Disease Topology Network Construction

The selected targets and pathways were summarized and imported into Cytoscape 3.7.2 software to construct a network of drug-target pathway disease relationships, followed by topological analysis using the Network Analyzer plugin.

#### 4.12.6. Molecular Docking Validation

Searching for the GRb1 structure in the PubChem database, the constituents were converted to mol2 format using Open Babel 3.1.1 software. The three-dimensional structure of the protein was retrieved from the PDB database, followed by desolvation using PyMOL software (https://www.pymol.org/). Subsequently, molecular docking visualization analysis of the protein and GRb1 was performed using AutoDock software (https://autodock.scripps.edu/).

## 5. Conclusions

In this study, we synthesized HA@GRb1@CS NPs and optimized the process to obtain the optimal preparation method: CS concentration of 2 mg/mL, TPP concentration of 2.3 mg/mL, CS to TPP mass concentration ratio of 1.5:1. The obtained nanoparticles exhibit a particle size of 126.4 nm, a zeta potential of 36.8 mV, and a PDI of 0.243. Characterization techniques including TEM, FTIR, XRD, and DSC confirmed the successful encapsulation of GRb1, demonstrating the successful preparation of HA@GRb1@CS NPs with stable quality and excellent performance for drug sustained release. In vitro cell experiments indicated the good safety profile of HA@GRb1@CS NPs with no cytotoxicity and significant enhancement of anti-inflammatory and antioxidant effects. Bioinformatics analysis suggested that the mechanism of HA@GRb1@CS NPs in treating CVDs may involve targeting signaling pathways such as STAT3, JUN, EGFR, and CASP3, regulating processes including apoptosis, autophagy, anti-lipid, and arterial vascular sclerosis.

## Figures and Tables

**Table 1:** PBD ANOVA and significance test (* *p* < 0.05, significant difference).

Source	Particle Size		Potential		EE		DL	
	*F*-Value	*p*-Value	*F*-Value	*p*-Value	*F*-Value	*p*-Value	*F*-Value	*p*-Value
Model	1.07	0.0030 *	1.02	0.5025	9.84	0.0120 *	5.31	0.0436 *
A	3.21	0.0003 *	1.03	0.3576	3.58	0.0012 *	22.99	0.0049 *
B	10.74	0.0220 *	8.59	0.3663	3.59	0.8573	6.30	0.5265
C	5.49	0.0662	8.87	0.3657	8.77	0.0315 *	1.96	0.2208
D	6.38	0.4523	1.03	0.3558	5.90	0.7065	1.68	0.2521
E	5.93	0.0590	8.73	0.3660	6.08	0.6313	2.22	0.8874
F	2.41	0.1816	1.09	0.3449	6.23	0.0548	4.73	0.0817

**Table 2:** Results of BBD.

Group	X_1_	X_2_	X_3_	Particle Size/nm	Potential/mV	EE/%	DL/%	OD Value
1	0	+1	−1	144.1	30.2	68.01	14.46	0.6926
2	0	0	0	169.4	34.1	71.74	12.99	0.7476
3	0	+1	+1	193.8	34.3	60.13	9.85	0.4817
4	0	−1	−1	184.1	30.2	72.41	14.93	0.7043
5	0	0	0	157.6	34.8	75.69	14.44	0.8502
6	−1	+1	0	212.3	34.5	55.76	12.52	0.5992
7	0	−1	+1	183.1	32.6	53.68	9.00	0.3168
8	0	0	0	205.8	31.1	69.85	14.56	0.6929
9	0	0	0	235.0	34.7	61.47	10.22	0.5031
10	+1	0	+1	298.6	30.5	37.93	8.61	0
11	+1	0	−1	188.3	31.3	66.69	11.78	0.5921
12	−1	−1	0	204.0	34.4	63.33	11.21	0.5947
13	0	0	0	122.5	37.2	83.23	15.23	1.0000
14	+1	+1	0	191.9	30.1	63.24	10.21	0.4563
15	−1	0	−1	183.6	29.4	65.74	10.50	0.4729
16	+1	−1	0	435.2	25.1	77.65	14.56	0
17	−1	0	+1	210.3	29.3	60.99	11.17	0.4710

**Table 3:** BBD ANOVA and significance test (* *p* < 0.05, significant difference).

Source	Sum of Squares	Degrees of Freedom	*F*-Value	*p*-Value
model	0.9210	9	4.2943	0.0339 *
X_1_	0.1483	1	6.2236	0.0413 *
X_2_	0.0471	1	1.9773	0.2025
X_3_	0.1777	1	7.4582	0.0293 *
X_1_X_2_	0.0010	1	2.1413	0.1868
X_1_X_3_	0.0871	1	3.6549	0.0975
X_2_X_3_	0.0078	1	0.3272	0.5852
X_1_^2^	0.2749	1	11.5389	0.0115 *
X_2_^2^	0.0346	1	1.4528	0.2672
X_3_^2^	0.0598	1	2.5109	0.1571
residual	0.1668	7	/	/
lack of fit	0.0304	3	0.2976	0.8265
pure error	0.1364	4	/	/
total deviation	1.09	16	/	/
*R* ^2^	0.9467	/	/	/
CV%	2.86%	/	/	/

**Table 4:** Results of fitting the drug release kinetic equation for HA@GRb1@CS NPs.

Medium	Model	Equation	*R* ^2^	Medium	Model	Equation	*R* ^2^
pH5.0 PBS	zero level	Q = 12.9 + 1.7t	0.7559	artificial intestinal fluid	zero level	Q = 18.2 + 1.6t	0.6337
	first order	Q = 76.5[1 − exp(−0.09t)]	0.9874		first order	Q = 72.2[1 − exp(−0.15t)]	0.9942
	Higuchi	Q = 12.5t^1/2^ + 1	0.9133		Higuchi	Q = 13.2t^1/2^ + 1	0.8550
	Ritger–Peppas	Q = 13.2t^0.48^	0.9139		Ritger–Peppas	Q = 18.1t^0.40^	0.8753
pH7.4 PBS	zero level	Q = 16.0 + 1.8t	0.7343	artificial gastric fluid	zero level	Q = 19.9 + 1.7t	0.6614
	first order	Q = 83.5[1 − exp(−0.10t)]	0.9888		first order	Q = 77.6[1 − exp(−0.15t)]	0.9952
	Higuchi	Q = 14.1t^1/2^ + 1	0.9076		Higuchi	Q = 14.1t^1/2^ + 1	0.8641
	Ritger–Peppas	Q = 16.0t^0.46^	0.9122		Ritger–Peppas	Q = 19.6t^0.39^	0.9003

**Table 5:** Effect of different mass fractions of mannitol on HA@GRb1@CS NPs.

Mannitol/%	Particle Size/nm	Potential/mV	PDI	EE/%	DL/%
1	161.0 ± 3.2	22.3 ± 1.0	0.275 ± 0.003	72.38 ± 1.58	10.05 ± 0.52
2	144.4 ± 4.1	29.3 ± 0.5	0.281 ± 0.005	76.92 ± 1.99	12.62 ± 0.21
5	129.9 ± 1.4	33.1 ± 0.2	0.274 ± 0.006	80.18 ± 1.34	15.03 ± 0.49
10	192.3 ± 4.9	26.6 ± 1.9	0.495 ± 0.014	67.62 ± 1.52	8.93 ± 0.15
20	285.4 ± 9.3	43.2 ± 3.7	0.556 ± 0.017	73.57 ± 2.47	6.68 ± 0.35

**Table 6:** PBD experimental factor levels.

Level	A	B	C	D	E	F
+1	3 mg/mL	3 mg/mL	3:1	5 mg	0.3%	2 h
−1	2 mg/mL	1 mg/mL	1:1	3 mg	0.1%	1 h

**Table 7:** BBD experimental factor levels.

Level	X_1_	X_2_	X_3_
+1	2.5 mg/mL	3 mg/mL	3:1
0	2.0 mg/mL	2 mg/mL	2:1
−1	1.5 mg/mL	1 mg/mL	1:1

## Data Availability

All data are contained within the article.
